# Frequency matters: How successive feeding episodes by blood-feeding insect vectors influences disease transmission

**DOI:** 10.1371/journal.ppat.1009590

**Published:** 2021-06-10

**Authors:** Doug E. Brackney, Jacquelyn C. LaReau, Ryan C. Smith

**Affiliations:** 1 Center for Vector-Borne and Zoonotic Diseases, Department of Environmental Sciences, The Connecticut Agricultural Experiment Station, New Haven, CT, United States of America; 2 Department of Entomology, Iowa State University, Ames, IA, United States of America; University of Wisconsin Medical School, UNITED STATES

Hematophagy, the process of consuming and metabolizing blood, is an integral part of the arthropod vector life cycle. Blood feeding provides a nutrient-rich meal that is required for adult egg production and developmental progressions of immature life stages. This reliance on blood results in frequent feeding episodes every 2 to 4 days [1,2] that enable arthropod disease vectors to acquire and transmit pathogens of medical and veterinary importance.

Blood feeding represents a significant physiological event, where hematophagous arthropods ingest several times their body weight in blood. Consequently, they must be able to rapidly digest the blood meal and remove excess water to regain their mobility. In addition to digestion, numerous other processes including oogenesis [3], immune induction [4,5], and the remodeling and regeneration of midgut epithelial cells are activated [6], highlighting the global physiological impacts associated with blood meal acquisition.

Historically, a singular blood meal has been used to introduce pathogens into vector species to study aspects of vector competence (the ability of a vector to become infected and ultimately transmit a pathogen) in laboratory experiments. While informative, such studies fail to consider how additional blood meals may directly or indirectly impact pathogen infection despite evidence that many hematophagous vectors routinely acquire multiple blood meals in nature. *Aedes aegypti*, the primary vector of dengue virus and Zika virus, as well as a number of *Anopheles* spp. mosquitoes that are responsible for transmitting human malaria parasites, are highly anthropophilic and will frequently imbibe multiple blood meals within a single gonotrophic cycle (the time from blood meal acquisition to egg laying; approximately 3 days) [7–10]. In fact, it has been estimated that *Ae*. *aegypti* acquire approximately 0.7 human blood meals a day in nature [7]. This frequent and preferential acquisition of human blood is evolutionarily advantageous as it has been shown to provide a fitness advantage in form of increased survival, reproductive output, and cumulative net replacement [11]. The additive effect of frequent feeding and fitness gains makes these mosquito species extremely effective vectors [1]. This multiple feeding phenotype extends beyond mosquitoes and can be observed in other important insect vectors. For example, blood meal analysis of sandflies (*Phlebotomus* spp.) revealed that 28% of field-caught individuals contained remnants of blood from multiple host species [12,13]. Together, these data highlight the importance of feeding frequencies of mosquito and sandfly vectors in natural settings and the potential that these multiple feeding behaviors influence the success of pathogen infection and disease transmission.

Recent work across multiple vector–pathogen systems is now beginning to reveal the significance that additional noninfectious blood meals can have on vector–pathogen interactions and its impacts on vector-borne disease transmission. Here, we discuss recent work highlighting the impact of multiple blood meals on arbovirus dissemination and transmission, malaria parasite adaptation to its mosquito vector, and influence on *Leishmania* parasite development and transmission.

## Arboviruses

### Multiple feeding promotes arbovirus dissemination from the mosquito midgut

Upon acquisition of an infectious blood meal, virus particles enter the midgut and, if successful, establish infection of the gut epithelial cells prior to escape into the open circulatory system and subsequent invasion of the salivary glands [14]. The timing and success of escape are not uniform as viruses must traverse/bypass the basal lamina encasing the midgut. With a pore size of approximately 10 nm, the basal lamina allows for the free passage of nutrients and hormones but prevents much larger arboviruses (approximately 50 to 70 nm) from passing [15,16]. This begs the question, how do arboviruses escape?

**Fig 1:**
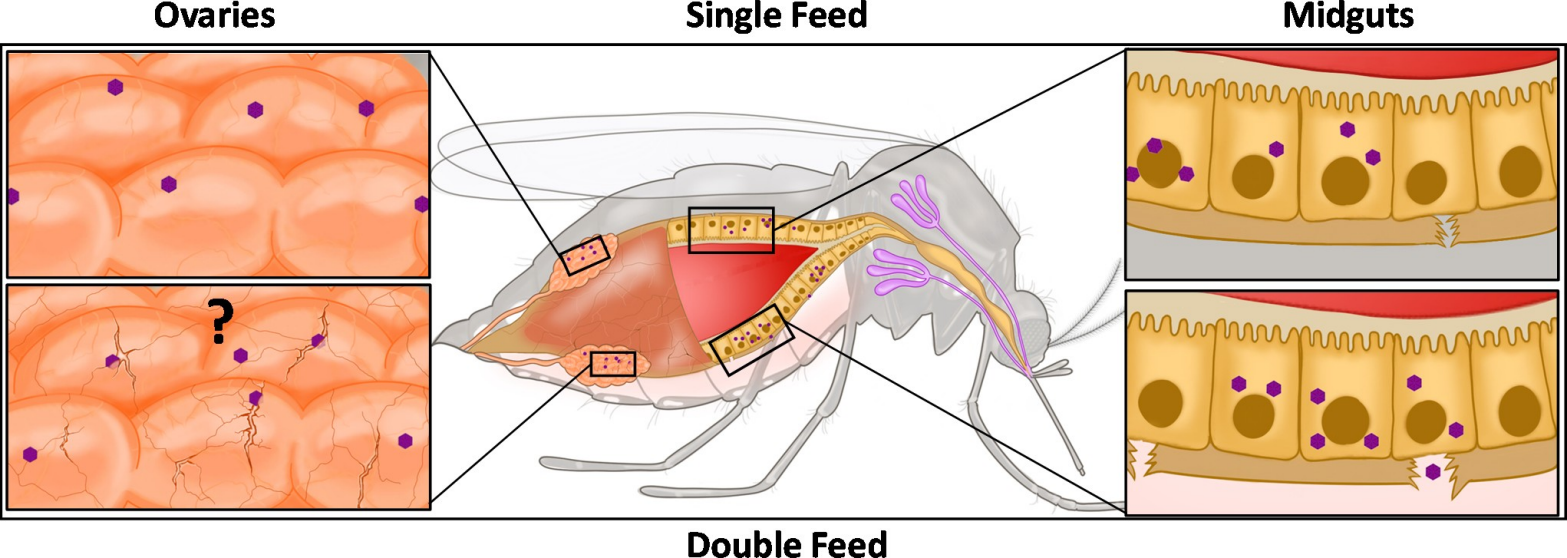
Multiple feeding episodes provide arboviruses opportunities to navigate anatomical barriers within mosquitoes. (Center) Cross section of *Ae*. *aegypti* mosquito infected with Zika virus under single-feed (top) and double-feed (bottom) scenarios. A dense network of proteoglycans, termed the “basal lamina,” provides support and protection to internal tissues such as the mosquito midgut (right inserts) and ovaries (left inserts). Due to pore size limitations, arboviruses cannot easily traverse these structures. However, additional blood feeding results in distention of the midgut tissue inducing micro-perforations in the underlying basal lamina facilitating virus passage to the hemolymph and access to secondary tissues such as the salivary glands. Similarly, blood meal acquisition induces oogenesis and expansion of the ovarian tissue. It is hypothesized that such disruptions in the ovarian basal lamina could provide viruses access to the developing eggs and infection of the progeny via vertical transmission.

To date, the kinetics of mosquito arbovirus infection has only primarily been examined following a single infectious blood meal and does not recapitulate the multiple feeding behaviors of mosquito vector species in the wild. Recent experiments in which mosquitoes were first offered an infectious blood meal and challenged 3 days later with a second noninfectious blood meal revealed that additional blood feeding-enhanced Zika virus, dengue virus, and chikungunya virus escape from the midgut, significantly shortening the duration between mosquito virus acquisition to transmission [17]. For years, it has been proposed that arboviruses escape through the basolateral surface of infected midgut cells when the integrity of the basal lamina has been compromised [18]. Examination of the basal lamina following a blood meal revealed damage associated with the distention of the midgut tissue [17,19,20], such that the current working model is that blood meal acquisition reduces the integrity of the midgut basal lamina thereby allowing virus dissemination out of the gut (Fig 1). Consequently, additional blood feeding would increase the likelihood that viruses traverse the basal lamina resulting in earlier and more efficient virus dissemination. Consequently, modelling these changes in virus dissemination kinetics revealed that the shortened extrinsic incubation period (EIP) (time from pathogen acquisition to transmission) associated with additional blood meals could significantly increase the basic reproductive number, *R*_0_ (the number of new infections arising from an infected mosquito) [17]. These findings demonstrate the real-world implications that multiple blood meals can have on the epidemiology and epidemic potential of mosquito-borne arboviruses.

### Invading the ovaries: How an additional blood meal increases vertical transmission

Arboviruses are predominantly transmitted horizontally (arthropod ↔ vertebrate), but when conditions are unfavorable (e.g., low numbers of susceptible hosts), arboviruses can also be maintained through other lower frequency mechanisms of transmission. Both field and laboratory studies have demonstrated that arboviruses from the genera *Flavivirus* and *Orthobunyavirus* can be maintained through vertical transmission (mother ↔ offspring) [21,22]. Interestingly, these studies have found that vertical transmission will only occur if an infected individual with a disseminated infection imbibes additional blood meals [23]. This observation is likely a function of the spatiotemporal kinetics of infection. Upon acquisition of an infectious blood meal, virus is typically confined to the midgut for at least 3 days before disseminating to secondary sites via the open circulatory system. However, oogenesis is typically completed within the first 3 days of feeding, suggesting that virus has not had time to infect, replicate, and escape the midgut and subsequently establish infection of the ovaries prior to deposition of the eggs. Despite a clear spatiotemporal element, it remains unclear how the virus invades the ovaries. While speculative, the mechanism of ovarian invasion might be intrinsically linked to blood feeding and oogenesis. Like the midgut, the ovaries are surrounded by a size-limiting basal lamina, which precludes arbovirus entry [24]. However, during oogenesis, the ovaries rapidly expand. Such expansion could result in damage to the basal lamina, thereby facilitating arbovirus invasion as was observed for virus escape from the midgut (Fig 1). Future studies will be needed to fully elucidate how arboviruses are able to invade the ovaries and be transmitted vertically.

## Malaria

### Differential immune recognition of malaria parasites following an additional blood meal

Shortly after the ingestion of a malaria-infected blood meal, *Plasmodium* parasites undergo a series of developmental progressions in the mosquito host that ultimately result in the production of a motile ookinete approximately 18 to 24 hours after feeding [25]. Following midgut invasion, successful ookinetes transition into oocysts on the basal surface of the midgut epithelium. There, *Plasmodium* oocysts reside in the interstitial space surrounded by the basal lamina where they mature and undergo sporogony over an approximately 2-week period [25]. As a result of this extended period of development, *Plasmodium* oocysts are the stage of the parasite that would most likely be influenced by an additional blood feeding.

When an additional blood meal is provided to *Plasmodium*-infected *Anopheles gambiae* 4 days postinfection to mimic the natural feeding cycle, a recent study identified that oocysts of the rodent malaria parasite, *Plasmodium berghei*, were significantly limited by an additional feeding, while no effect on the human malaria parasite, *Plasmodium falciparum*, was determined [26]. While previous studies have demonstrated that mosquito complement does not contribute to *Plasmodium* oocyst killing in traditional single-feed experiments [27,28], additional feeding and subsequent degradation of the midgut basal lamina enables thioester-containing protein 1 (TEP1) recognition and killing of *P*. *berghei* oocysts [26]. Moreover, similar experiments in *An*. *gambiae* infected with human malaria parasites demonstrate that TEP1 does not recognize *P*. *falciparum* oocysts [26], suggesting that successfully invading *P*. *falciparum* ookinetes that initially evaded TEP1 and mosquito complement recognition [29–31] are similarly protected during the oocyst stage. However, these differences in oocyst immune recognition between *Plasmodium* species are still unknown, warranting future study.

Of interest, when an additional feeding was provided later in oocyst development (8 days postinfection), there were no impacts on oocyst numbers for either species [26], suggesting that as oocysts mature, they become immune privileged through modifications to the parasite surface once sporogony has initiated. Together, these studies argue that additional feeding in *Plasmodium*-infected mosquitoes significantly contribute to mosquito vector competence and the selection of parasite species to evolve with its mosquito host.

### Influence of blood feeding on *Plasmodium* oocyst growth and malaria parasite transmission

**Fig 2:**
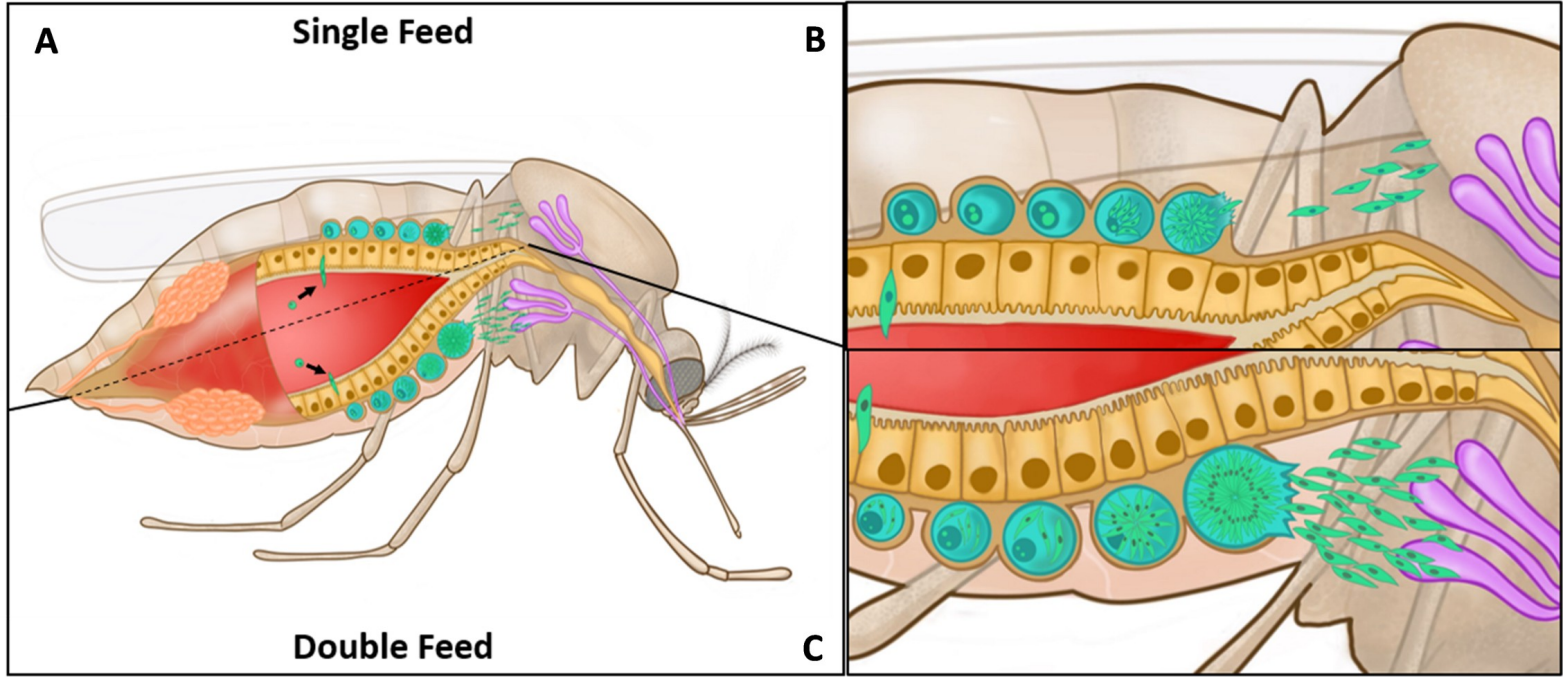
Additional blood meals increase the transmission potential of malaria-infected mosquitoes. **(A)** Cross section of an *An*. *gambiae* mosquito with the developmental stages of the human malaria parasite *P*. *falciparum* under single-feed (top) and double-feed (bottom) scenarios. Compared to a single blood meal **(B)**, *P*. *falciparum*–infected mosquitoes provided with an additional blood meal increase oocyst growth rates and sporogony, resulting in faster rates of sporozoite salivary gland invasion and reducing the time required for malaria parasite transmission **(C)**.

In addition to the impacts on immune recognition of *Plasmodium* oocysts associated with an additional blood meal, several recent studies have demonstrated that an additional feeding can also influence parasite growth [26,32,33]. In mosquitoes receiving an additional blood meal, *P*. *falciparum* oocysts display increased size [26,32,33] as a result of the added nutrient resources provided with feeding, which overcome nutritional dormancy to promote parasite growth [33]. The resulting increased growth translates into an accelerated sporozoite invasion of the mosquito salivary glands, reducing the EIP and increasing the likelihood of malaria transmission [32] (Fig 2).

Comparable experiments examining the effects of additional feeding on *P*. *berghei* oocyst growth suggest that the timing of an additional meal during parasite development may influence the ability of the parasite to utilize these added resources. Oocyst size was unchanged when provided an additional blood meal at 4 days postinfection [26], yet *P*. *berghei* oocysts display an increase in growth when an additional feed is delayed and provided later in parasite development (7 days postinfection) [33]. Evidence suggests that an additional feeding overcomes a dormancy-like state in *P*. *berghei* similar to *P*. *falciparum* [33], yet further experiments are required to determine any potential influence of additional blood meal on vectorial capacity.

## Leishmania

### Blood feeding promotes *Leishmania* parasite replication and differentiation

Evidence suggests that sequential blood meals by the sandfly vector are also integral to the transmission of *Leishmania* parasites, enabling the establishment of infection through parasite amplification at multiple stages of infection [34]. Naturally acquired infections (through feeding on a vertebrate host) have fewer parasite numbers than traditional laboratory infections (where an artificial infected blood meal is provided), where *Leishmania* parasites are either lost or result in a low intensity of infection in the absence of an additional blood meal [34]. This is in contrast to sandflies that take an additional blood meal following a naturally acquired infection, which encourages the establishment of infection, a significant increase in metacyclic promastigote numbers, and results in more efficient *Leishmania* transmission [34].

This is supported through laboratory-based studies (using an artificial infection) demonstrating that an additional blood meal provided at 6 days postinfection promotes further parasite replication, resulting in increased parasite numbers, higher percentages of metacyclic promastigotes, and the production of a larger haptomonad parasite sphere (HPS) enhancing the blockage of the stomodeal valve [34]. Experiments in both Old and New World sandfly vectors demonstrate that this increase in parasite numbers is a conserved response to subsequent blood feeding in diverse *Leishmania* parasite: sandfly vector systems [34].

**Fig 3:**
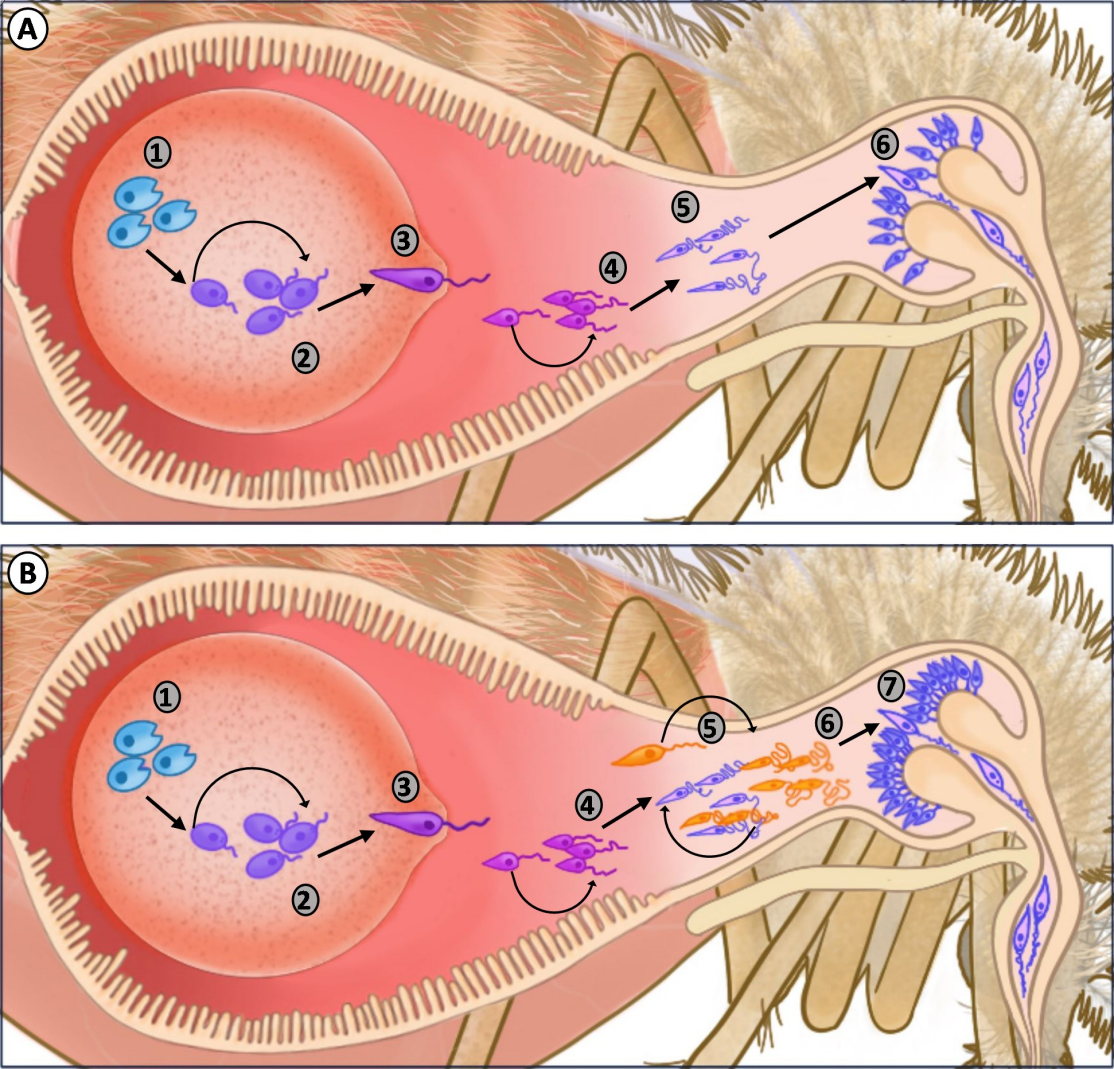
Multiple feeding by infected sandflies promotes *Leishmania* parasite differentiation, increasing the efficiency of transmission. **(A)** Depiction of *Leishmania* parasite development in a sandfly following a single blood meal under laboratory conditions. Immotile amastigotes (1) emerge from ingested macrophages and transform into the replicative procyclic promastigote form (2). Subsequently, replication slows, and the procyclic promastigotes transform into highly motile nectomonad promastigotes (3) and exit the peritrophic-encased blood bolus. Within the anterior portion of the midgut lumen, the parasites further develop into the replicative leptomonad promastigote form (4) and the (5) haptomonad promastigotes which attach the stomodeal valve. The leptomonads transform into infectious metacyclic promastigotes and migrate to the stomodeal valve where they can be regurgitated and transmitted during subsequent feeding events. **(B)** When sandflies with a mature infection take an additional blood meal, metacyclics undergo dedifferentiation into retroleptomonads (6), while earlier stages of parasite development (steps 1–5) have already matured. The production of retroleptomonads in response to an additional blood meal enables further parasite replication before differentiation back into metacyclic promastigotes, thus amplifying the number of infectious metacyclic (7) parasites that increase the potential for transmission.

Furthermore, when an additional blood meal is provided at 12 days postinfection (representing a second additional blood feeding), metacyclic promastigotes (a previously believed terminally differentiated parasite stage) undergo conversion into retroleptomonad promastigotes [34] (Fig 3). This newly described stage denoted by a large cell body, short flagella, and low motility enables the further amplification of parasite numbers in the sandfly midgut before differentiation back into metacyclic promastigotes, dramatically increasing metacyclic numbers and blockage of the stomodeal valve to further promote *Leishmania* parasite transmission [34] (Fig 3).

Based on these results and the propensity of sandflies to take a blood meal every 5 to 6 days, Serafim and colleagues [34] propose that multiple sequential blood meals are required for *Leishmania* transmission under natural field conditions, demonstrating the important epidemiological impacts of additional blood feeding on the incidence of leishmaniasis.

## Concluding remarks

In summary, recent studies have begun to highlight the integral yet previously unexplored mechanisms by which vector blood feeding contributes to the transmission of vector-borne diseases. While at present, evidence has only been provided for mosquitoes and sandflies, the influence of multiple blood meals on both viral and parasitic pathogens suggests that arthropod feeding behaviors may influence a wide range of vector–pathogen interactions. Moreover, evidence suggests that parasite or virus infection can manipulate vector blood-feeding behaviors [2,35–40], further highlighting the influence of additional blood meals on the transmission of veterinary and medically important diseases.
